# Supplementary material for: Raman microscopy-based quantification of the physical properties of intracellular lipids
Source: Commun Biol. 2021 Oct 8;4:1176. doi: 10.1038/s42003-021-02679-w (PMC8501034; doi:10.1038/s42003-021-02679-w)
Supplement: Supplementary file 2 — Supplementary Information [file 42003_2021_2679_MOESM2_ESM.pdf]

Supplementary information

Raman microscopy-based quantification of the physical properties of intracellular lipids

Masaaki Uematsu[1,2,*], Takao Shimizu[1,3]

[1]Department of Lipid Signaling, National Center for Global Health and Medicine, 1-21-1 Toyama, Shinjuku-ku, Tokyo 162-8655, Japan.

[2]Department of Lipidomics, Graduate School of Medicine, The University of Tokyo, 7-3-1 Hongo, Bunkyo-ku, Tokyo 1130-0033, Japan.

[3]Institute of Microbial Chemistry, 3-14-23 Kamiosaki, Shinagawa-ku, Tokyo 141-0021, Japan.

[*]Correspondence should be addressed to Masaaki Uematsu (uematsu@m.u-tokyo.ac.jp).

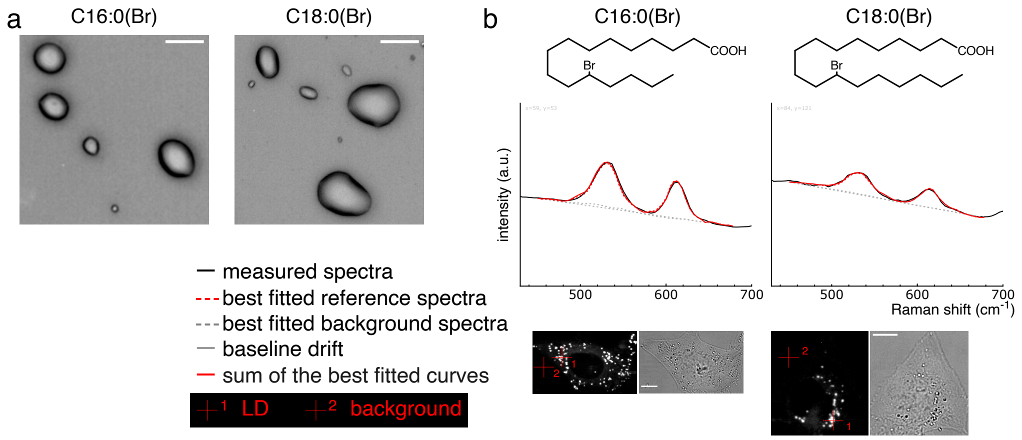

Fig. S1. Comparison of Raman spectra of unsaturated fatty acid between *in vivo* and *in vitro*. a Bright field image of C16:0(Br) and C18:0(Br). Indicated fatty acids were dropped on to CaF_2 glass substrate, and directly measured by Raman microscopy. **b** HeLa cells treated with indicated Br-labeled fatty acids (30 µM) for 24 h were fixed and observed using Raman microscopy. Representative LD spectra (represented by red crosshair 1 in the bottom images) are displayed with black lines, and the results of best fitted *in vitro* spectra, background spectra, baseline drifts, and their summations are shown in dashed red lines, dashed gray lines, dashed straight gray lines, and solid red lines, respectively. Spectra at red crosshair 2 in the bottom images were used as the background spectra for the fittings. Measurements of multiple cells and samples resulted in the similar results. Scale bars indicate 10 µm.

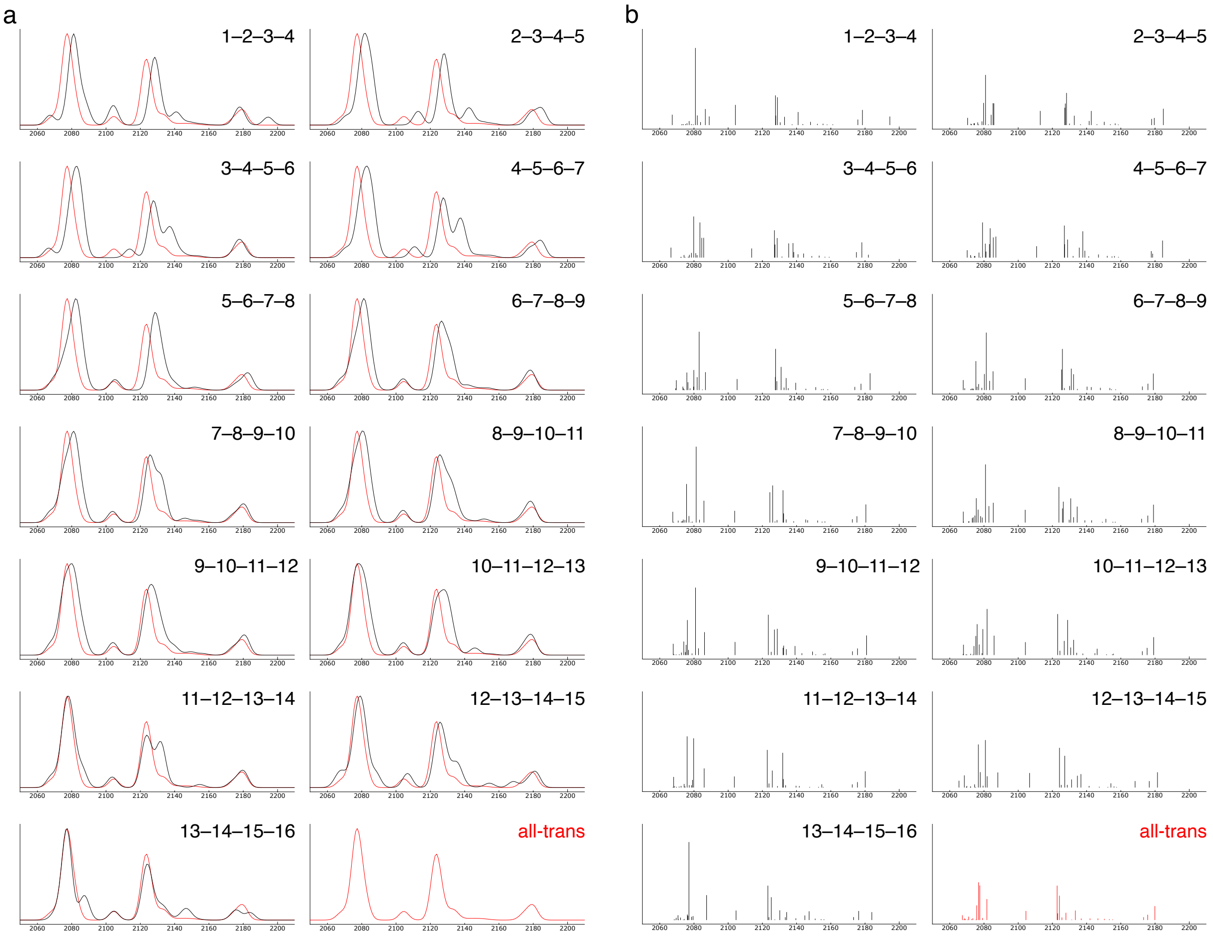

Fig. S2. Simulated Raman spectra and Raman activity of C16:0(d31) with one gauche conformation using DFT. a The four sequential numbers at the top right of each graph indicate the positions of four sequential carbon atoms in gauche conformation. Red lines displayed in all graphs depict the simulated spectra of C16:0(d31) with all-trans conformations. Simulated Raman spectra with no gauche conformation (all-trans) and with one gauche conformation at 7–8–9–10 consecutive carbons are shown as representative in Fig. 2. **b** The Raman activity of corresponding Raman spectra shown in (**a**).

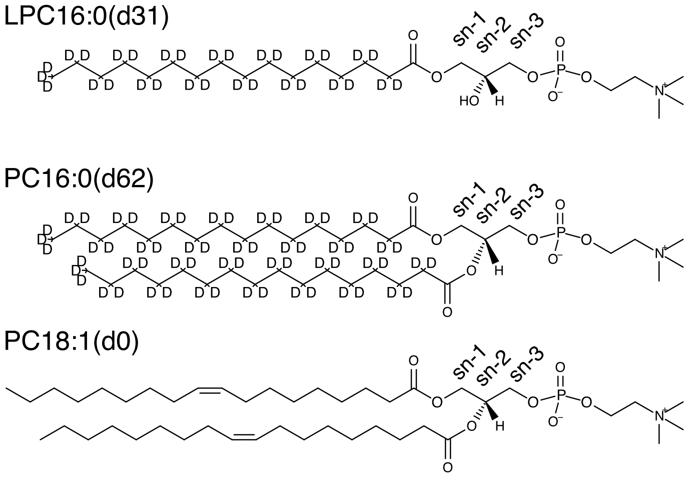

Fig. S3. Structure of LPC16:0(d31), PC16:0(d62), and PC18:1(d0). The numbers following the letters “sn-” indicate the position of carbon atoms in the glycerol backbone.

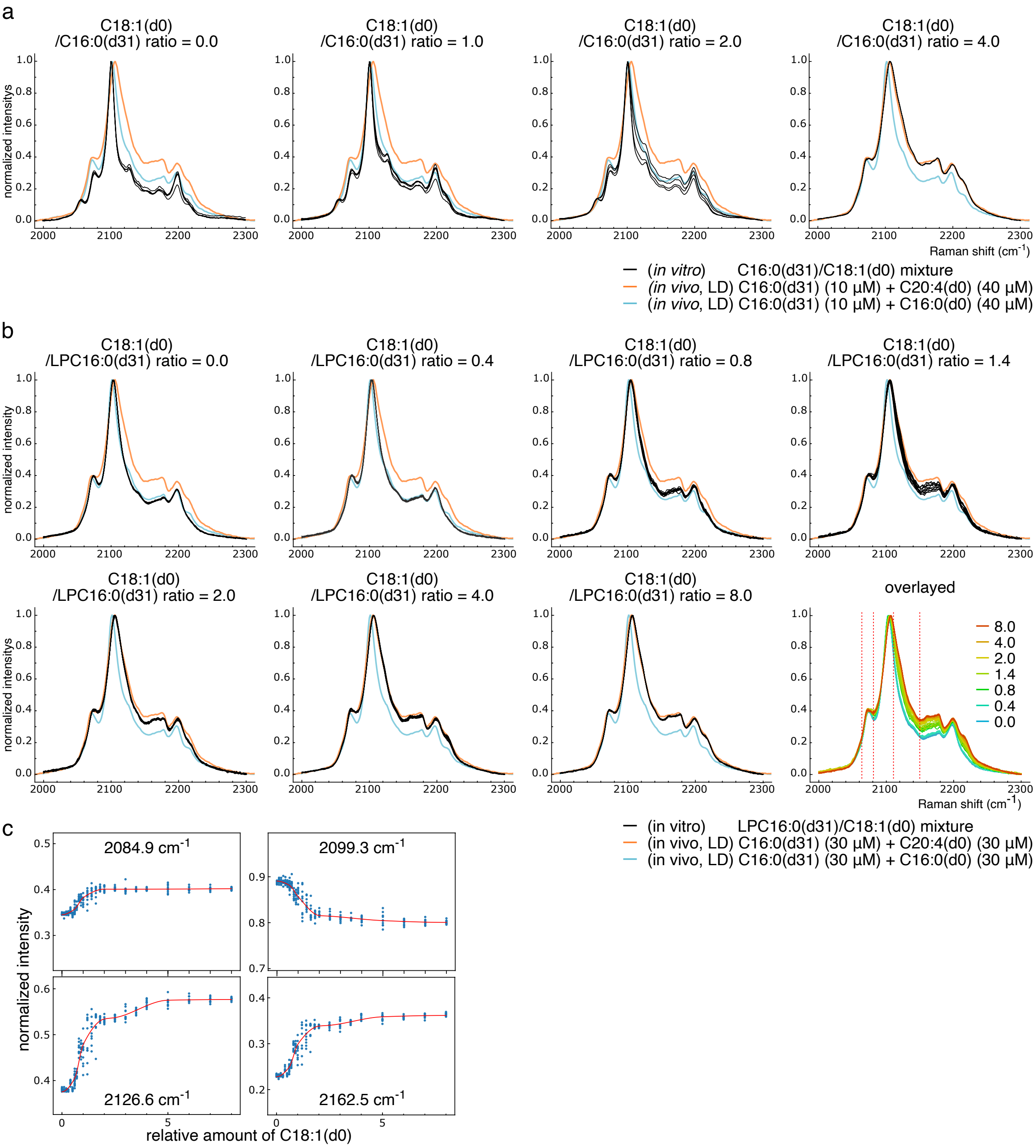

Fig. S4. *In vitro* spectra of C16:0(d31) and LPC16:0(d31). a *In vitro* spectra of C16:0(d31) mixed with C18:1(d0). Fatty acids were mixed with the indicated ratio, and Raman spectra were then measured. For each condition, 3 to 5 spectra were measured from different locations in the sample, each displayed in the same graph with black lines. To show the similarity of *in vitro* spectra to *in vivo* spectra, *in vivo* spectra of LDs from Fig. 3a are displayed using blue and orange lines. **b** *In vitro* spectra of LPC16:0(d31) mixed with C18:1(d0). Lipids were mixed with the indicated ratio, and Raman spectra were then measured. For each condition, 9 or 10 spectra were measured from different locations in the sample and are displayed in the same graph with black lines (seven graphs except for the

bottom right). To show the similarity of *in vivo* spectra to *in vitro* spectra, *in vivo* spectra of LDs from Fig. 3a are displayed using blue and orange lines. The bottom right graph depicts an overlaid graph of the other seven graphs. **c** Representative transitions of intensity at fixed Raman shift values. Transitions of intensity at four Raman shift values; 2084.9, 2099.3, 2126.6, and 2162.5 cm^{-1} (dashed four vertical red lines in the bottom right graphs of [**b**]) are displayed using blue dots. Red lines indicate interpolated curves using PCHIP algorithm.

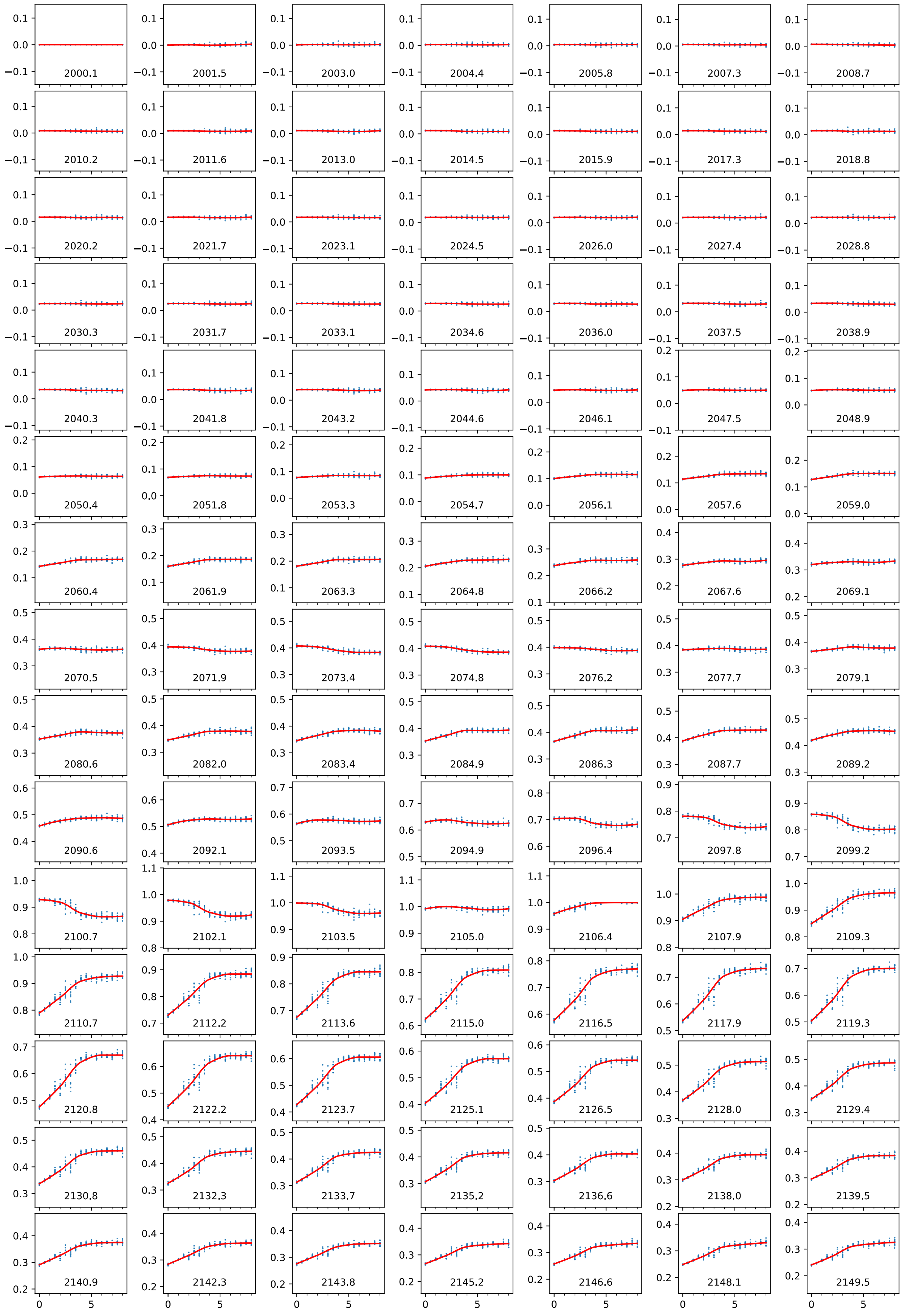

Fig. S5.

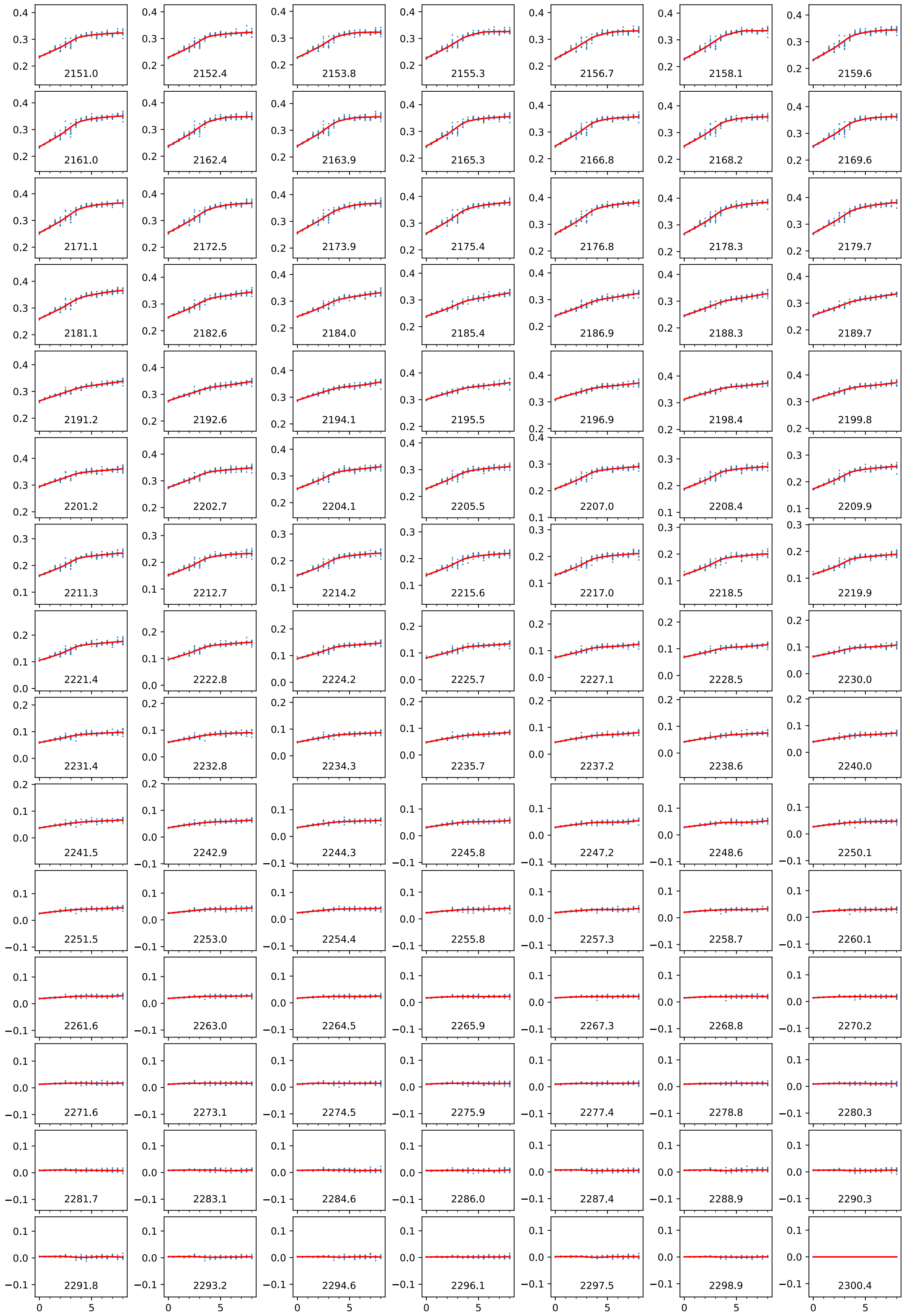

Fig. S5 (continued).

Fig. S5. Transition of intensity at each data point with a fixed Raman shift value.
Transitions of intensity at each Raman shift value (cm^{-1}) are displayed using blue dots in each graph. Red lines indicate interpolated curves using PCHIP algorithm (see Materials and Methods). X- and Y-axes indicate the PC18:1(d0)/PC16:0(d31) ratio and the relative intensity at each Raman shift, respectively.

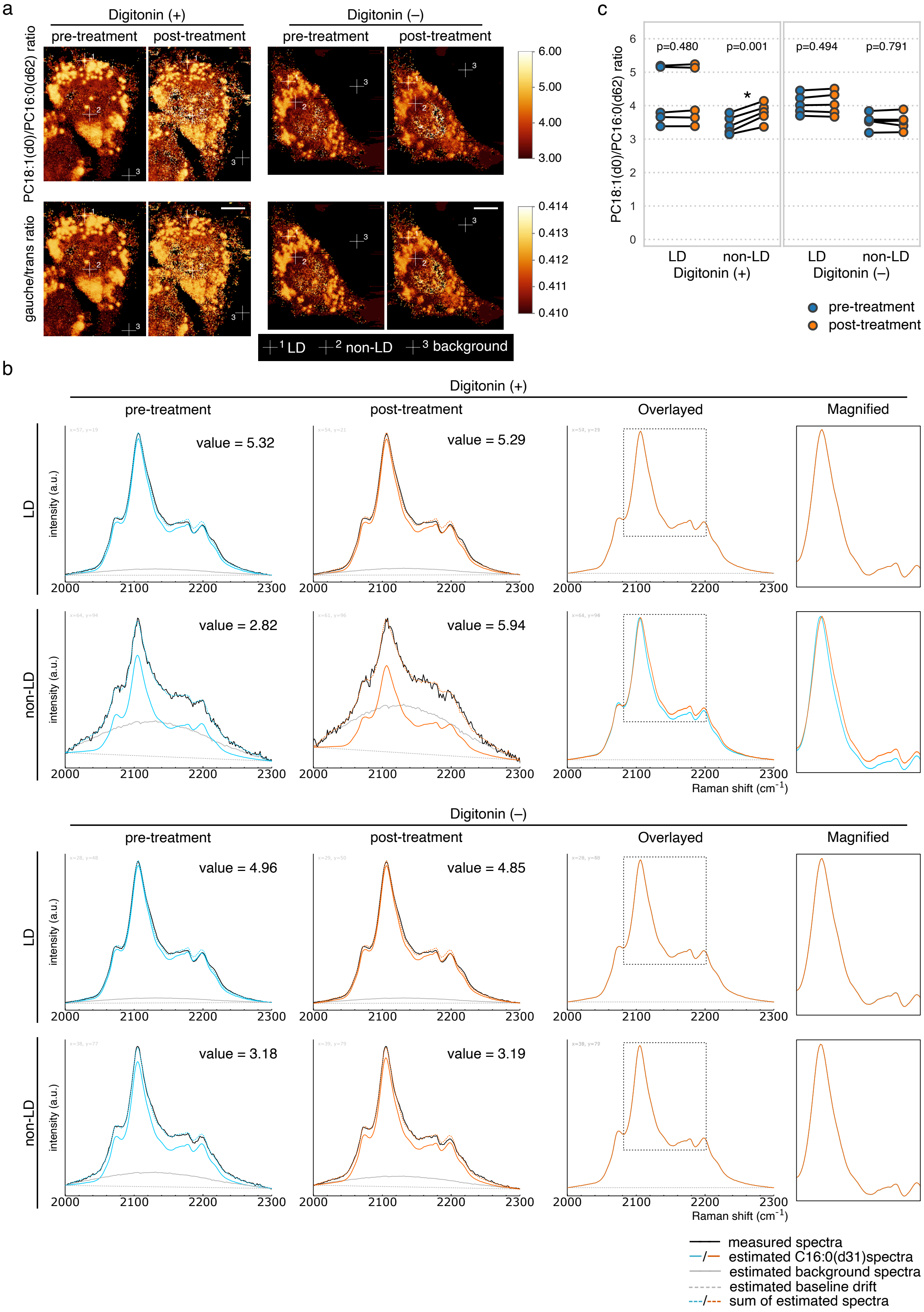

Fig. S6. Disruption assay altered the physical properties of lipids in non-LD regions. a The representative results of the Raman imaging of disruption assay. HeLa cells were treated

with the mixture of C16:0(d31) and C20:4(d0) (30 μM each) for 24 h and then measured with Raman microscopy (pre-treatment). Immediately after the first Raman imaging, the same cells were measured again in the presence or in the absence of 50 μg/ml of digitonin (post-treatment). The re-constructed PC18:1(d0)/PC16:0(d62) and gauche/trans ratio images are displayed. Scale bars indicate 10 μm. **b** Representative Raman spectra at each pixel indicated with white crosshairs 1 and 2 in (**a**). Results of the best-fitted spectra to the measured spectra (black lines) are displayed with the estimated C16:0(d31) spectra, estimated background spectra, estimated baseline drift, and the summation of them (solid-colored lines, solid gray lines, dashed gray lines, and dashed colored lines, respectively). The average of the background spectra (displayed with red crosshair 3 in [**a**]) was used for the fittings. The comparisons of the estimated C16:0(d31) spectra are shown as overlaid graphs after correcting for the baseline drift. Enlarged views of the area framed by the dotted squares are displayed on the right graphs. **c** Quantification of the averaged PC18:1(d0)/PC16:0(d62) ratio in LD and non-LD regions. The results from five cells from five independent dishes are displayed, and the asterisk indicates the significant difference (p<0.05) by the two-sided paired t-test with 95% confidence interval.

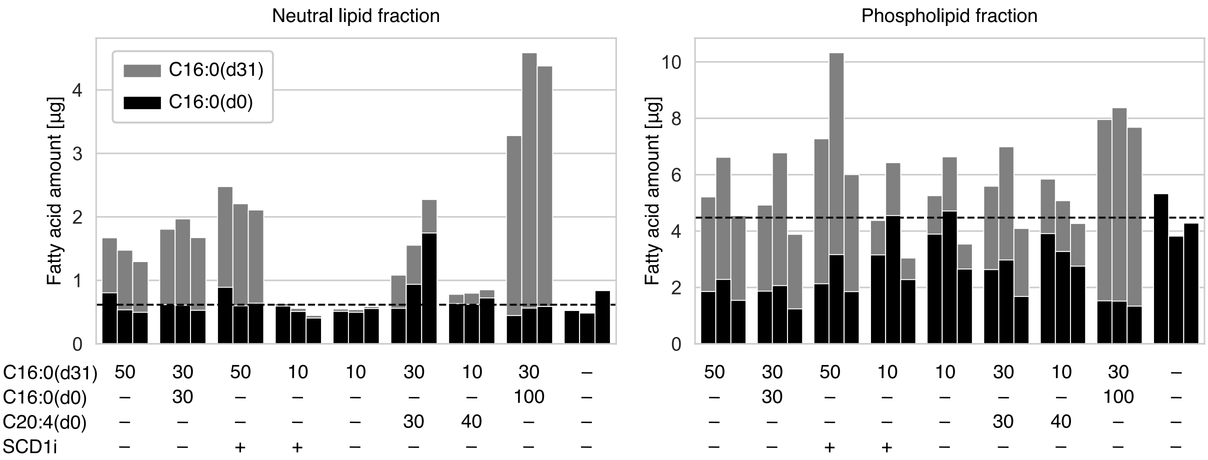

Fig. S7. Quantification of C16:0 amount for each condition. HeLa cells were treated with the same conditions as Figs. 5f, g, and h, in addition to the control condition. The amount of C16:0(d0) and C16:0(d31) in the neutral lipid and the phospholipid fractions per 12.25 cm^2 area (corresponding to an area of 3.5 cm dish) were measured by GC-MS. The average amounts of C16:0(d0) in the rightmost control conditions for each fraction are displayed as the dashed black lines. The results from three independent dishes are shown for each condition.

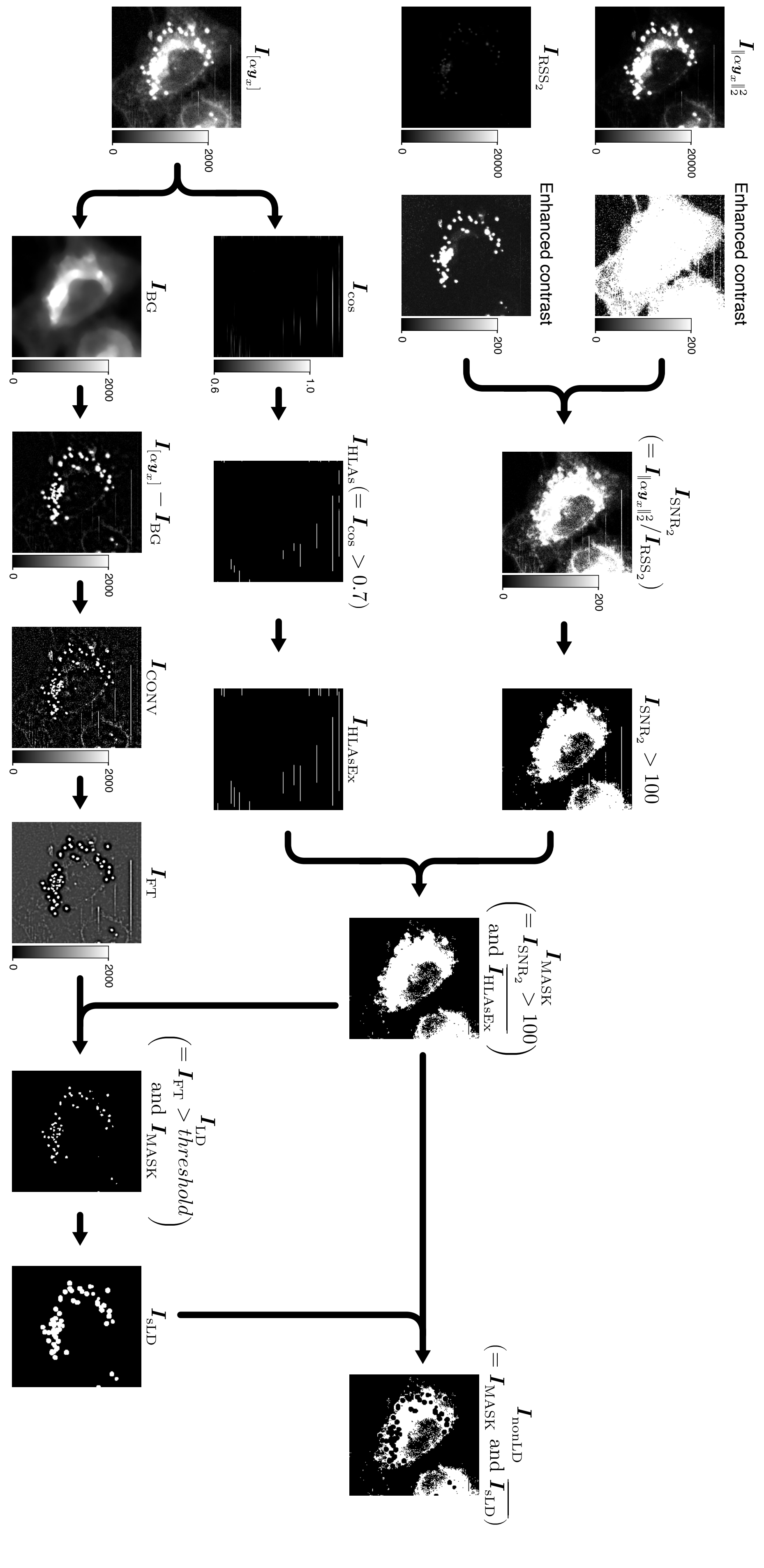
$I_{\|\alpha y_x\|_2^2}$
Enhanced contrast
I_{RSS_2}
Enhanced contrast
I_{SNR_2} $(= I_{\|\alpha y_x\|_2^2} / I_{RSS_2})$
$I_{SNR_2} > 100$
$I_{[\alpha y_x]}$
$I_{\cos}$
$I_{HLAs} (= I_{\cos} > 0.7)$
I_{HLAsEx}
I_{MASK} $\left(= I_{SNR_2} > 100 \text{ and } \overline{I_{HLAsEx}}\right)$
I_{BG}
$I_{[\alpha y_x]} - I_{BG}$
I_{CONV}
I_{FT}
I_{LD} $\left(= I_{FT} > \textit{threshold} \text{ and } I_{MASK}\right)$
I_{sLD}
I_{nonLD} $\left(= I_{MASK} \text{ and } \overline{I_{sLD}}\right)$

Fig. S8. Procedure for image quantification. Image of the square sum ($\boldsymbol{I}_{\|\alpha \boldsymbol{y}_x\|_2^2}$), the residual sum of squares ($\boldsymbol{I}_{\mathrm{RSS}_2}$), and the area under the curve ($\boldsymbol{I}_{[\alpha \boldsymbol{y}_x]}$) of the spectrum from C–D stretch were generated as the byproduct of converting Raman hyperspectral data into the PC18:1(d0)/PC16:0(d62) ratio image. By dividing $\boldsymbol{I}_{\|\alpha \boldsymbol{y}_x\|_2^2}$ by $\boldsymbol{I}_{\mathrm{RSS}_2}$, a signal to noise ratio (SNR) image was created ($\boldsymbol{I}_{\mathrm{SNR}_2}$), and pixels satisfying $\boldsymbol{I}_{\mathrm{SNR}_2} > 100$ were used as a mask to omit data with low SNR. Separately, $\boldsymbol{I}_{[\alpha \boldsymbol{y}_x]}$ was processed for two aims: the detection of horizontal line artifacts (HLAs), and the definition of LD regions. To detect HLAs, cosine similarity was calculated against a horizontal binary image (31 by 3), with only the middle row having an intensity of 1 ($\boldsymbol{I}_{\cos}$). Next, pixels satisfying $\boldsymbol{I}_{\cos} > 0.7$ were defined as HLAs ($\boldsymbol{I}_{\mathrm{HLAs}}$), followed by the horizontal extension of $\boldsymbol{I}_{\mathrm{HLAs}}$ by 10 pixels ($\boldsymbol{I}_{\mathrm{HLAsEx}}$). By combining pixels with $\boldsymbol{I}_{\mathrm{SNR}_2} > 100$ and $\boldsymbol{I}_{\mathrm{HLAsEx}}$, $\boldsymbol{I}_{\mathrm{MASK}}$ was defined and used as a mask in subsequent analyses. To define LD regions, the median background ($\boldsymbol{I}_{\mathrm{BG}}$) was subtracted from $\boldsymbol{I}_{[\alpha \boldsymbol{y}_x]}$, followed by convolutional processing with custom kernel ($\boldsymbol{I}_{\mathrm{CONV}}$) and bandpass filter processing using Fourier transformation ($\boldsymbol{I}_{\mathrm{FT}}$). Finally, a threshold defined using the modal intensity of $\boldsymbol{I}_{\mathrm{FT}}$ was applied to define LD regions ($\boldsymbol{I}_{\mathrm{LD}}$). Non-LD regions were defined as the area inside $\boldsymbol{I}_{\mathrm{MASK}}$ that is not included in swelled LD regions ($\boldsymbol{I}_{\mathrm{sLD}}$), which were created by swelling $\boldsymbol{I}_{\mathrm{LD}}$ by two pixels. $\bar{\boldsymbol{I}}$ represents the inverted binary image of $\boldsymbol{I}$.
