# Supplementary material for: Raman microscopy-based quantification of the physical properties of intracellular lipids
Source: Commun Biol. 2021 Oct 8;4:1176. doi: 10.1038/s42003-021-02679-w (PMC8501034; doi:10.1038/s42003-021-02679-w)
Supplement: Supplementary file 3 — Description of Additional Supplementary Files [file 42003_2021_2679_MOESM3_ESM.pdf]

## Description of Supplementary Files

**File name:** Supplementary Data 1
**Description:** Source data of the relationship between the gauche/trans ratio and the PC18:1(d0)/PC16:0(d62) ratio generated by in silico simulation (corresponds to Figure 4D).

**File name:** Supplementary Data 2
**Description:** Source data of quantified PC18:1(d0)/PC16:0(d62) and gauche/trans ratios for LD and non-LD regions, and area ratio of LD to LD + non-LD regions (corresponds to Figure 5F, G, and H).

**File name:** Supplementary Data 3
**Description:** Source data of the spectral transitions of the mixture of LPC16:0(d31) and C18:1(d0) (corresponds to blue dots in Figure S4C). Of note, the first row and column the table indicate the relative C18:1(d0) amount and the Raman shift, respectively. The other field of the table indicates the relative intensity at indicated C18:1(d0) amount and the Raman shift.

**File name:** Supplementary Data 4
**Description:** Source data of the spectral transitions used to generate the reference spectra shown in Figure 4C (corresponds to blue dots in Figure S5). Of note, the first row and column the table indicate the relative PC18:1(d0) amount and the Raman shift, respectively. The other field of the table indicates the relative intensity at indicated PC18:1(d0) amount and the Raman shift.

**File name:** Supplementary Data 5
**Description:** Data set used to generate the reference spectra shown in Figure 4C (corresponds to the red curves in Figure S5). The red curves shown in Figure S5 pass through the coordinates listed in the corresponding cell in this table. The equations of the red curves between the two coordinates are computed as the cubic functions by equations 3, 4, 5, and 6 in the main manuscript. Of note, the first row and column of the table indicate the relative PC18:1(d0) amount and the Raman shift, respectively. The other field of the table indicates the relative intensity at indicated PC18:1(d0) amount and the Raman shift.

**File name:** Supplementary Data 6
**Description:** Source data of quantified PC18:1(d0)/PC16:0(d62) ratio of LD and non-LD region for pre and post of disruption assay (corresponds to Figure S6C).

**File name:** Supplementary Data 7
**Description:** Source data of the quantification of C16:0 amount for each condition (corresponds to Figure S7).
